# Transcriptional Regulation of Müllerian Inhibiting Substance (MIS) and Establishment of a Gonadal Somatic Cell Line Using *mis-GFP* Transgenic Medaka (*Oryzias latipes*)

**DOI:** 10.3389/fendo.2020.578885

**Published:** 2020-09-29

**Authors:** Toshiaki Kawabe, Hiroyuki Kariya, Seiji Hara, Tsuyoshi Shirozu, Eri Shiraishi, Koki Mukai, Takashi Yazawa, Seiya Inoue, Takeshi Kitano

**Affiliations:** ^1^Department of Biological Sciences, Graduate School of Science and Technology, Kumamoto University, Kumamoto, Japan; ^2^ARK Resource Co., Ltd., Kumamoto, Japan; ^3^Department of Biochemistry, Asahikawa Medical University, Asahikawa, Japan

**Keywords:** MIS, germ cell, gonadal somatic cell, medaka (*Oryzias lapites*), hybridoma

## Abstract

In vertebrate germ cell differentiation, gonadal somatic cells and germ cells are closely related. By analyzing this relationship, it has recently been reported in mammals that primordial germ cells (PGCs), induced from pluripotent stem cells and germline stem cells, can differentiate into functional gametes when co-cultured *in vitro* with fetal gonadal somatic cells. In some fish species, differentiation into functional sperm by reaggregation or co-culture of gonadal somatic cells and germ cells has also been reported; however, the relationship between gonadal somatic cells and germ cells in these species is not well-understood. Here, we report the transcriptional regulation of Müllerian inhibiting substance (MIS) and the establishment of a gonadal somatic cell line using *mis-GFP* transgenic fish, in medaka (*Oryzias latipes*)—a fish model which offers many advantages for molecular genetics. MIS is a glycoprotein belonging to the transforming growth factor β superfamily. In medaka, *mis* mRNA is expressed in gonadal somatic cells of both sexes before sex differentiation, and MIS regulates the proliferation of germ cells during this period. Using luciferase assays, we found that steroidogenic factor 1 (SF1) and liver receptor homolog 1 (LRH1) activate medaka *mis* gene transcription, probably by binding to the *mis* promoter. We also report that *mis-GFP* transgenic medaka emit GFP fluorescence specific to gonadal somatic cells in the gonads. By fusing Sertoli cells from transgenic medaka with a cell line derived from medaka hepatoma cancer, we produced a hybridoma cell line that expresses gonadal somatic cell-specific markers, including Sertoli and Leydig cell markers. Moreover, embryonic PGCs co-cultured with the established hybridoma, as feeder cells, proliferated and formed significant colonies after 1 week. PGCs cultured for 3 weeks expressed a germ cell marker *dnd*, as well as the meiotic markers *sycp1* and *sycp3*. Thus, we here provide the first evidence in teleosts that we have successfully established a gonadal somatic cell-derived hybridoma that can induce both the proliferation and meiosis of germ cells.

## Introduction

Germ cells are the only cell lineage that contributes to the next generation. Germ cell differentiation is unique and precise, encompassing a dramatic differentiation from primordial germ cells (PGCs) to gametes. This differentiation is caused by interactions between gonadal somatic cells and germ cells. The gonadal somatic cells important in this role are the Sertoli cells (supporting cells) and Leydig cells (stromal cells) in males, and the granulosa cells and theca cells in females. These cells secrete important growth factors and steroids for germ line development (1, 2). Interactions between gonadal somatic cells and germ cells have been studied using *in vitro* cultivation, especially in mammals. Indeed, several recent reports have shown that germline stem cells can be cultured and can differentiate into functional gametes in mammals (3–5). Furthermore, studies on spermatogenesis using organ culture and *in vitro* culture have been reported in various species of fish, such as medaka (*Oryzias latipes*) (6), Japanese eel (*Anguilla japonica*) (7), zebrafish (*Danio rerio*) (8, 9), tilapia (*Oreochromis niloticus*) (10), and rainbow trout (*Oncorhynchus mykiss*) (11). These studies have shown that the mechanisms of differentiation and development of gonads, including germ cells in fish, can be directly evaluated by *in vitro* cultivation methods. Further evaluation of these relationships awaits the establishment of gonadal somatic cell lines and analysis of expression factors. In fish, the somatic cell lines have been established in some species; these were derived from cancers, natural mutation by long-term cultivation, or the addition of carcinogenic substances (12–14). In practice, cells can be immortalized via several methods; for example, immortalizing mutations can be induced in target cells, and hybridomas can be produced using established immortalized cell lines. Notably, in the generation of monoclonal antibodies, antibody-producing B cells and myeloma cells are immortalized by cell fusion to produce hybridomas (15). Therefore, cell fusion could be used to immortalize gonadal somatic cells; however, to date no gonadal somatic hybridomas have been reported, due to a lack of selective media for screening and cloning.

Müllerian inhibiting substance (MIS), also known as anti-Müllerian hormone, is a glycoprotein belonging to the transforming growth factor β superfamily, which is involved in the regulation of growth and differentiation in mammals (16). In mice, MIS shows sexually dimorphic expression patterns. It is expressed in males during sex differentiation, where it is first detected in the Sertoli cells of the testis shortly after the initial expression of the testis-determining gene *Sry* (17); expression then persists after regression of the Müllerian ducts (18). In females, ovarian *Mis* mRNA expression is first detected in granulosa cells 6 days after birth and remains low throughout the reproductive life of the mouse (18). Analysis of the transcriptional regulation of *Mis* in mice has indicated that Ad4 binding sites are required for *MIS* promoter activity *in vivo* and *in vitro* (19). It is also known that the Ad4 site binds the nuclear receptor steroidogenic factor 1 (SF1) and liver receptor homolog 1 (LRH1) to regulate gene transcription (20–22). Therefore, *mis* expression is likely to be driven by SF1 and LRH1 in gonadal somatic cells such as Sertoli cells and granulosa cells in mammals.

In teleosts, reports about the *mis* promoter are available for six different species: Japanese flounder (*Paralichthys olivaceus*), medaka, zebrafish, European seabass (*Dicentrarchus labrax*), Atlantic salmon (*Salmo salar*) and rainbow trout (23). All *mis* promoter sequences show potential Ad4 binding sites and the predicted binding motifs for GATA- and POU-class transcription factors (23). Previously, an electrophoretic mobility shift assay showed that both SF1 and LRH1 bind to a potential Ad4 binding site of *mis* promoter in Japanese flounder (24); however, the detailed transcriptional regulation of teleost *mis* remains unclear.

Medaka is an excellent vertebrate model organism for studies of sex determination and differentiation (25–28). A small laboratory fish with an XX/XY sex determination system, it has advantages such as a short generation time, small genome size, and several useful strains are available (29). Additionally, transgenesis, knockdown techniques, and genome editing using clustered regularly interspaced short palindromic repeats (CRISPR)/CRISPR-associated protein 9 have been established (30–32). Medaka is therefore a valuable vertebrate model for the analysis of the molecular genetics of various biological phenomena, including embryonic development and sex differentiation. During sex differentiation in medaka, *mis* mRNA is expressed in the gonadal somatic cells of both sexes (33) and MIS regulates germ cell proliferation during early gonadal differentiation (31). If we can generate the transgenic medaka that visualize *mis* expression, it may be used for screening gonadal somatic hybridomas.

Here, to elucidate the transcriptional regulation of medaka *mis in vitro* and *in vivo*, we first investigated whether medaka SF1 and LRH1 activate *mis* transcription using a luciferase assay and transgenic techniques. Next, we investigated whether *mis-GFP* transgenic medaka emit GFP fluorescence that is specific to the gonadal somatic cells. Additionally, to immortalize the gonadal somatic cells, we established a novel hybridoma, which had gonadal somatic cell-like gene expression, by fusing GFP-positive gonadal somatic cells with a medaka cell line. Finally, to evaluate whether the hybridoma is useful for culturing PGCs, we examined the proliferation and differentiation patterns of PGCs by co-culturing PGCs with our novel hybridoma.

## Materials and Methods

### Animals

We used the FLFII medaka strain for our transgenic and hybridoma studies (34). The FLFII strain allows the identification of genotypic sex by the appearance of leucophores 2 days post-fertilization (dpf), that is, before the onset of sex differentiation. The *olvas-DsRed* transgenic FLFII medaka line, which enables the visualization of germ cells by DsRed fluorescence (25), was used in the culturing experiment. Fish embryos were maintained in ERM (17 mM NaCl, 0.4 mM KCl, 0.27 mM CaCl_2_ 2H_2_O, 0.66 mM MgSO_4_, pH 7) at a water temperature of 26°C in a 14-h light and 10-h dark cycle. Developmental stages of the embryos were determined as described previously (35).

### Construction of Vectors

Fragments containing 5′-flanking regions (3.1 and 0.8-kb) of the putative start codon (ATG) of the medaka *mis* gene were amplified from the Hd-rR medaka genome by PCR with appropriate primers; primers are listed in Supplementary Table 1. The *mis-GFP* vectors were constructed by inserting these fragments into KpnI/BamHI sites of pEGFP-1 (Clontech); *mis-luciferase* reporter vectors were constructed by inserting these fragments into KpnI/XhoI sites of PicaGene Basic Vector 2 (Nippon Gene Co. LTD., Tokyo, Japan). SF1 and LRH1 expression vectors were constructed by ligating the medaka *sf1* and *lrh1* cDNAs amplified by PCR with appropriate primers listed in Supplementary Table 1, into EcoRI/XhoI sites of pcDNA3.1 (Invitrogen Corp., Carlsbad, CA), respectively.

### Generation of Transgenic Lines

The *mis-GFP* vectors were injected into the cytoplasm of medaka embryos at the one-cell stage using a Nanoject II (Drummond Scientific Co., Broomall, PA) as previously described (25). The injected embryos were grown to adulthood and about 10 adults were pair-mated to non-transgenic medaka to obtain F1 embryos. GFP-positive heterozygous embryos were selected by observing GFP fluorescence under a fluorescence stereomicroscope (MZFL III, Leica Microsystems, Wetzlar, Germany), grown to adulthood, and then crossed with each other. Homozygous offspring were confirmed by backcrossing to non-transgenic fish and used for the experiments. Fluorescent images were captured using a BZ-9000 BioRevo fluorescence microscope (Keyence Co., Osaka, Japan), or a Fluoview FV10i confocal microscope (Olympus, Tokyo, Japan).

### *In situ* Hybridization

Nine-dpf embryos were fixed in 4% (w/v) paraformaldehyde in 10 mM phosphate-buffered saline (PBS) at 4°C overnight, then embedded in paraffin and sectioned serially at 5 μm thickness. *GFP* and *mis* antisense RNA probes were *in vitro* transcribed from pT7Blue T-vectors (Novagen) into which *GFP* or *mis* cDNA had been inserted. *In situ* hybridization was performed using a digoxigenin (DIG)-labeled *GFP* or *mis* antisense RNA probe as previously described (31).

### Quantitative Real-Time PCR (qRT-PCR)

Total RNA was extracted from the medaka cell lines, embryos, and adult gonads using ISOGEN (Nippon Gene) and reverse-transcribed at 42°C for 30 min using an RNA PCR kit (Applied Biosystems, Foster, CA) as previously described (26). qRT-PCR was performed using the SYBR Green I Master Mix (Roche, Mannheim, Germany) on a LightCycler 480 (Roche) with appropriate primers; primers are listed in Supplementary Table 1. The cycle conditions were as follows: preheating PCR was carried out at 95°C for 5 min, followed by 45 cycles of 95°C for 10 s, 59°C for 10 s, and 72°C for 10 s. The copy number of each target gene was calculated based on *elongation factor-1*α *(ef1*α*)*. All experiments were performed in triplicate at minimum.

### Cell Culture

Hepa-E1 cells (derived from the eel hepatocyte cell line) were cultured in phenol-red free E-RDF medium (Kyokuto Pharmaceutical Co. Ltd., Tokyo, Japan) supplemented with 5% charcoal/dextran treated fetal calf serum (HyClone, Logan, UT) at 28°C. OLHE-131 cells derived from medaka hepatoma cancer (12), were purchased from RIKEN BRC (RCB#0187) and acclimatized in Leibovitz's L-15 medium (Invitrogen) by transferring 15 times for 6 months. The medaka testis cells were dispersed from adult *mis-GFP* transgenic testis using 0.25% Trypsin-EDTA (Thermo Fisher Scientific, Waltham, MA, USA), 1 mg/ml collagenase type I (Wako Pure Chemical Corporation, Osaka, Japan), and 10 mg/ml dispase type II (Godo Shusei Co. Ltd., Tokyo, Japan) treatments at 28°C for 15 min, removed impurities by centrifugation and 40 μm cell strainer (Corning Inc., NY, USA), and then cultured in Leibovitz's L-15 medium supplemented 10% fetal bovine serum (FBS, GE Healthcare, IL, USA) until cell fusion.

### Transfection and Luciferase Assay

Hepa-E1 cells were plated in 24-well plates 24 h before transfection. Cells were transfected with 240 ng of the *mis-luciferase* reporter, the SF1-expression, LRH1-expression or empty vector (control), and 40 ng of pRL-TK *Renilla* luciferase normalization vector (Nippon Gene) using Lipofectamine^TM^ reagent (Thermo Fisher Scientific) as described previously (36). The luciferase assay was performed using the Dual-Luciferase Reporter Assay System (Promega, Madison, WI) and a MiniLumat LB9506 (Berthold, Pforzheim, Germany). All transfections were performed in tetraplicate.

### Cell Fusion and Cloning

Cell fusion of OLHE-131 cells and *mis-GFP* transgenic testis cells was performed by the PEG fusion technique as previously described (37). In brief, the OLHE-131 cells and testis cells were dispersed by Trypsin-EDTA, mixed gently with 50% PEG 1500 in Dulbecco's phosphate buffered saline (D-PBS, Nacalai Tesque, Kyoto, Japan.) for 2 min, serially diluted with L-15 medium, and then cultured in L-15 medium supplemented 10% FBS until cell confluence in 96-well plates. Single GFP-positive cells were picked by the glass capillaries after Trypsin-EDTA treatment, cultured until cell confluence in 96-well plates, and then repeated twice. After culture, single GFP-disappeared cells were cloned by transferring twice because GFP fluorescence disappeared gradually in the hybridomas. Cell number was determined using a hemocytometer, and relative cell proliferation was calculated by dividing the final number of cells by the number of primary cells.

### PGC Cultivation

*olvas-DsRed* transgenic embryos at 3–4 dpf and 6–8 dpf were used for PGC cultivation. Gonadal regions, including PGCs, were dissociated from dechorionated embryos with 28-30-gauge needles and dispersed by Trypsin-EDTA treatment for 10–20 min at 28°C. The dispersed PGCs (5–30 cells) were co-cultured with and without OLHE-131 or FOT-02 in L-15 medium (basic medium) supplemented with 1% GlutaMAX ^TM^ Supplement (Thermo Fisher Scientific), 10% FBS, and 2.5% common carp serum (ARK Resouce Co., Ltd, Kumamoto, Japan). For co-culture, cell proliferation in confluent OLHE-131 and FOT-02 cells was stopped by treatment with 10 μg/ml mitomycin C for 4 h at 28°C; cells were then washed four times with sterilized D-PBS. PGC states were confirmed visually by the expression of red fluorescence at 1, 2, and 3 weeks after culture. Plating efficiency was assessed by dividing the number of colonizing germ cells by the number of plated cells.

### Statistics

Experimental results were tested using Levene's test for homogeneity of variance. Data were analyzed by Student's *t*-test or by one-way ANOVA followed by Tukey's multiple comparison test, using SPSS statistics 20 (IBM Corp., Armonk, NY).

## Results

### Characterization of the Medaka *mis* Promoter, and Its Activation by SF1 and LRH1

**Figure 1:**
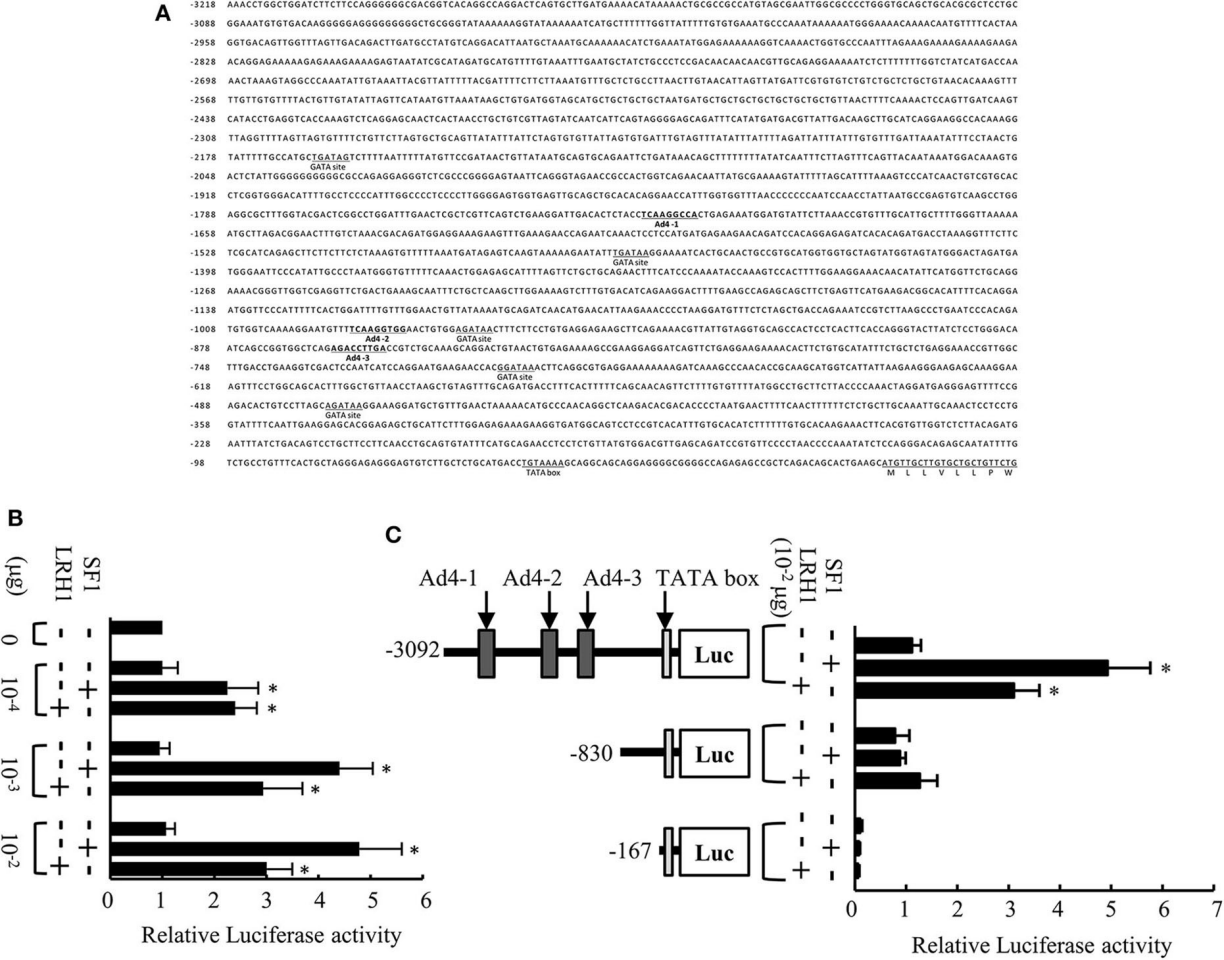
Analysis of the medaka *mis* promoter. **(A)** Sequence of the 5′-flanking region of the medaka *mis* gene. Three Ad4 sites are indicated in bold. **(B)** Transcriptional activity of the 3.1-kb *mis* promoter in Hepa-E1 cells. **(C)** Transcriptional activity of deletion constructs of the *mis* promoter in Hepa-E1 cells. Relative luciferase (Luc) activity was calculated based on the value of activity in cells transfected without the expression plasmids. Vertical bars indicate means (SEM) of tetraplicates. Significant differences from controls transfected with plasmids lacking steroidogenic factor 1 (SF1) and liver receptor homolog 1 (LRH1) are indicated by *p < 0.05.

The 5′-flanking region of the medaka *mis* gene contains a putative TATA box, five GATA sites, and three potential Ad4 sites (Ad4-1, 5′-TCAAGGCCA-3′; Ad4-2, 5′-TCAAGGTGG-3′; Ad4-3, 5′-AGACCTTGA-3′) within 3,218 bp upstream of the translation initiation codon (Figure 1A). To investigate whether SF1 and LRH1 activate medaka *mis* gene transcription *in vitro*, we performed luciferase transfection assays using Hepa-E1 cells. We found that SF1 and LRH1 significantly induced luciferase activity via the 3.1-kb *mis* promoter fragment (Figure 1B), but not via the *mis* promoter fragments (0.8 and 0.2-kb) that lack the three Ad4 sites (Figure 1C).

### Generation and Characterization of *mis-GFP* Transgenic Medaka

**Figure 2:**
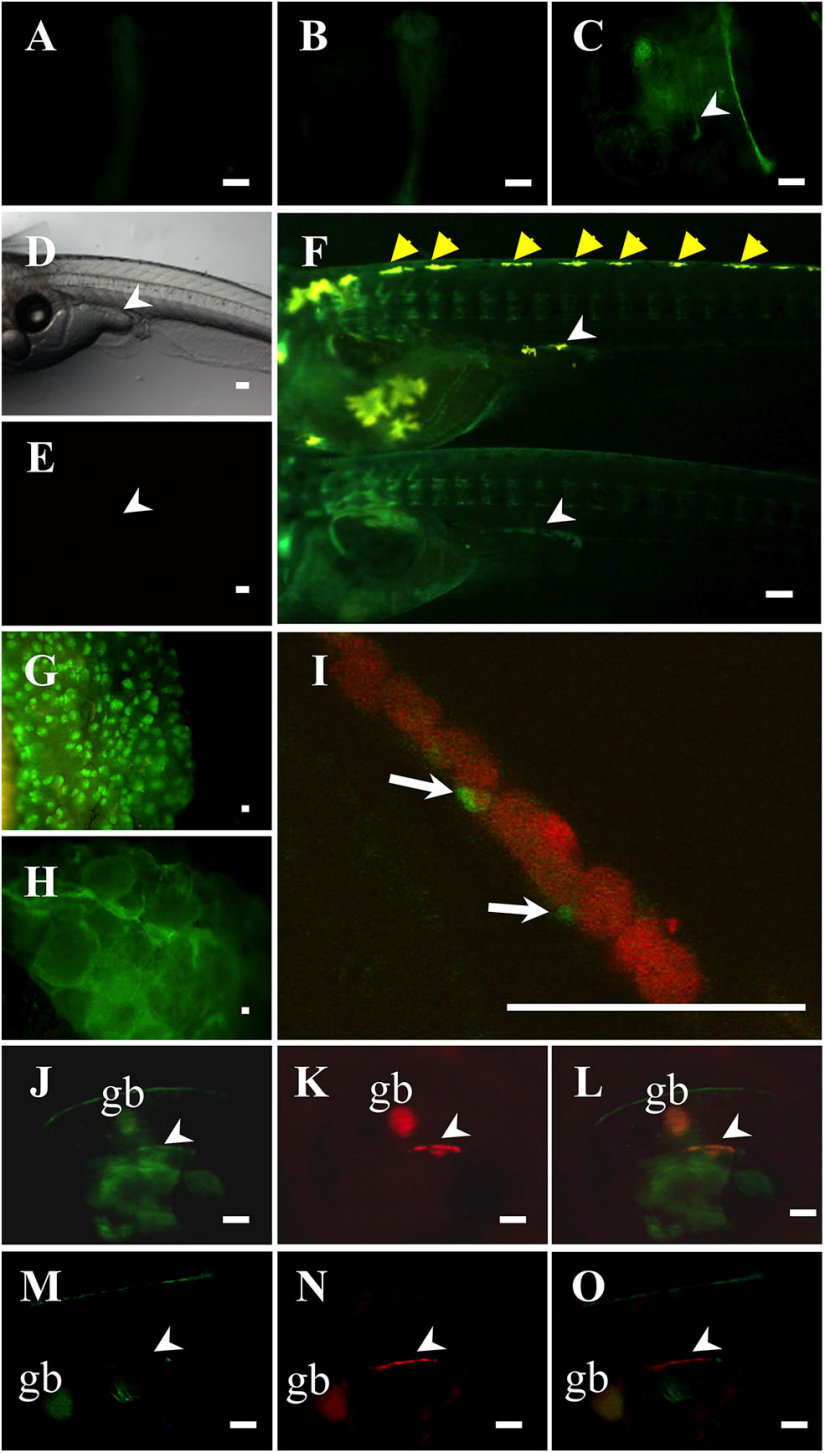
Characterization of *mis-GFP* transgenic medaka. GFP expression in *mis (–3.1-kb)-GFP* transgenic medaka at 1-dpf **(A)**, 2-dpf **(B)**, and 4-dpf **(C)**. Non-transgenic medaka at 9-dpf in bright field **(D)** and in dark field **(E)**. GFP expression in *mis (*−*3.1-kb)-GFP* transgenic medaka at 9-dpf (**F**: upper, XY; lower, XX; at hatching), testis **(G)**, and ovary **(H)**. **(I–L)** *mis (–3.1-kb)-GFP/olvas-DsRed* double transgenic medaka at 9-dpf. The expression of GFP **(J)** and DsRed **(K)**, and a merged image **(I,L)** are shown. **(M–O)** *mis (*−*0.8-kb)-GFP/olvas-DsRed* double transgenic medaka at 9-dpf. The expression of GFP **(M)** and DsRed **(N)**, and a merged image **(O)** are shown. White arrow: GFP-positive cell; White arrowhead: gonadal region; yellow arrowhead: leucophore; gb, gallbladder. Scale bar: 100 μm.

To investigate whether the Ad4 sites of the medaka *mis* promoter are indispensable for *mis* gene transcription *in vivo*, we established two of each *mis-GFP* transgenic medaka, using constructs containing either 3.1 or 0.8-kb fragments of the *mis* promoter fused to GFP. Two of each transgenic medaka had the similar GFP expression patterns (data not shown). In the *mis (*−*3.1-kb)-GFP* transgenic medaka, GFP expression was observed throughout the whole body at 1 and 2 dpf (Figures 2A,B). From 4 dpf (stage 33), GFP was detected highly in the gonadal region of both sexes (i.e., before sex differentiation); GFP was also detected in the dorsomedial region (Figures 2C–F). In the adult gonads, GFP expression in the testis was scattered, while it was observed in a lattice-like pattern in the ovary (Figures 2G,H). Notably, in *mis (*−*3.1-kb)-GFP/olvas-DsRed* transgenic medaka, GFP was localized in the gonadal somatic cells surrounding germ cells (Figures 2I–L); however, in the *mis (*−*0.8-kb)-GFP/olvas-DsRed* transgenic medaka that possess the promoter lacking the Ad4 sites, GFP expression in the gonadal region was rarely observed (Figures 2M–O).

**Figure 3:**
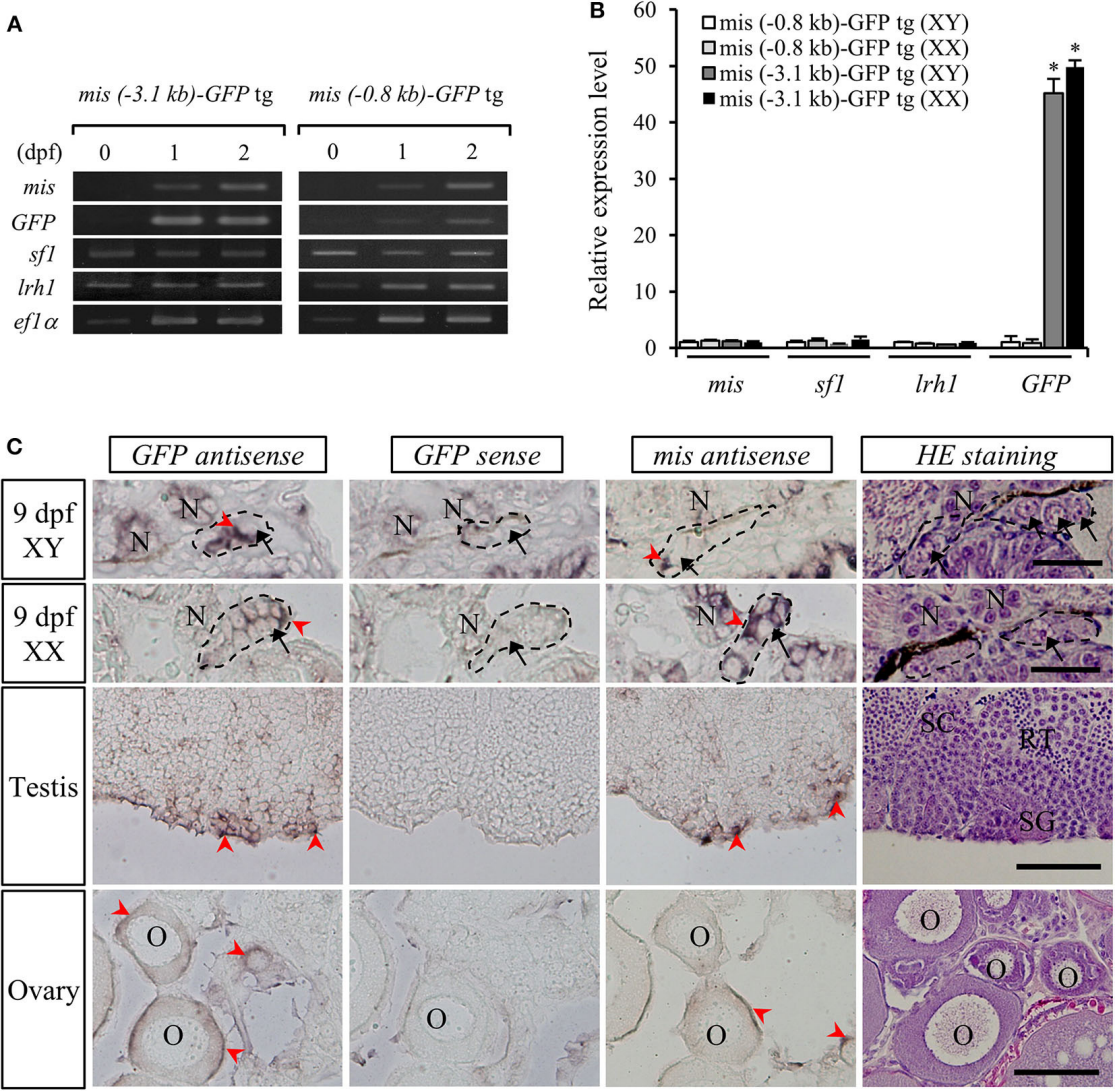
Gene expression in *mis-GFP* transgenic (tg) medaka. RT-PCR analysis of *mis, GFP, sf1*, and *lrh1* mRNAs in the embryos at 0-dpf, 1-dpf, 2-dpf **(A)** and 9-dpf **(B)**. *p < 0.05. *In situ* hybridization of *mis* and *GFP* transcripts and hematoxylin/eosin (HE)-stained sections in gonads of both sexes **(C)**. Arrow, germ cell; arrowhead, positive cell; SC, spermatocyte; SG, spermatogonium; RT, round spermatid; O, oocyte; N, nephridium. Scale bar: 100 μm.

The relative expression levels of *mis, GFP, sf1*, and *lrh1* mRNAs in embryos (10 pooled samples) of both *mis-GFP* lines were confirmed by RT-PCR analysis. This revealed that *sf1* and *lrh1* mRNAs were detectable from 1 dpf, prior to *mis* and *GFP* mRNAs being observed (Figure 3A). On the day of hatching (stage 39), there were no differences in the relative expression levels of *mis, sf1*, and *lrh1* between the two strains or between sexes; however, *GFP* expression was higher in both sexes of the line generated with the 3.1-kb *mis* promoter fragment (Figure 3B).

To confirm the localization of *GFP* mRNA in the gonads of *mis (–3.1-kb)-GFP* transgenic medaka, we performed *in situ* hybridization with DIG-labeled *GFP* or *mis* antisense RNA probes. At 9 dpf (stage 39), both *mis* and *GFP* mRNAs were detected in the gonadal somatic cells surrounding the germ cells of both sexes (Figure 3C). In adult gonads, they were localized in the Sertoli cells surrounding the spermatogonia of the testis and in the granulosa cells of the ovary (Figure 3C). There was no signal in the gonads when a *GFP* sense RNA probe was used (Figure 3C).

### Fusion and Cloning of Gonadal Somatic Cells

**Figure 4:**
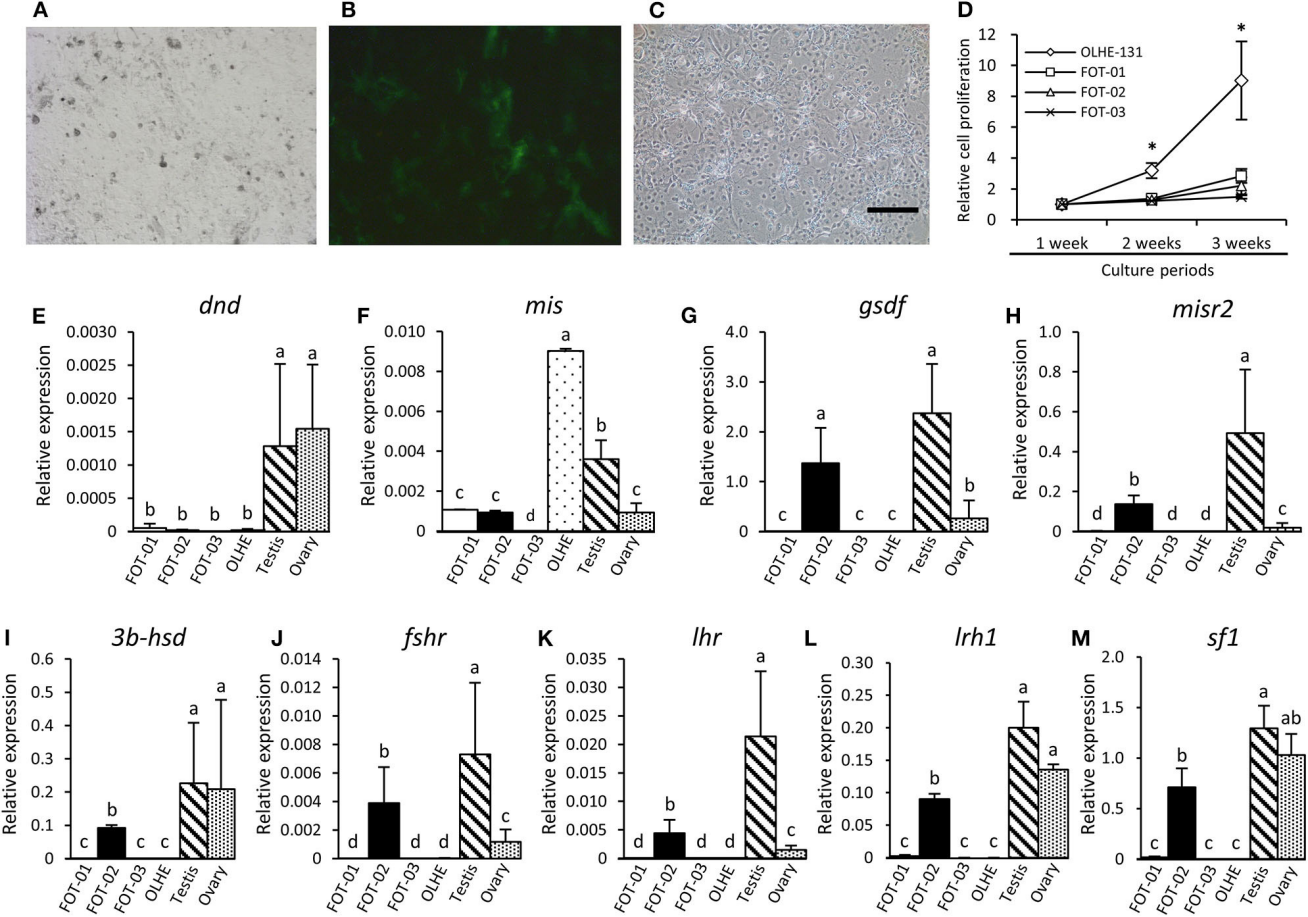
Characteristics of the hybridoma lines derived from Sertoli cells of *mis*-GFP transgenic testes. GFP fluorescence in primary cultured testis cells from *mis-GFP* transgenic medaka in bright field **(A)** and in dark field **(B)**. Established hybridoma, FOT-02 **(C)**. Comparison of growth rate between established hybridomas and OLHE-131 **(D)**. *p < 0.05. Confirmation of mRNA expression in the hybridomas cultured for 3 weeks by qRT-PCR. The germ cell marker *dnd* **(E)** was expressed only in the testis and ovary. The gonadal somatic cell markers: *mis* **(F)**, *gsdf* **(G)**, *misr2* **(H)**, *3b-hsd* **(I)**, *fshr* **(J)**, *lhr* ***(*K)**, *lrh1***(L)**, and *sf1***(M)** were significantly expressed in FOT-02. Relative expression was calculated based on the expression of *ef1*. Scale bar: 200 μm. Vertical bar: mean ± standard error of triplicates; a, b, c, d, and ab: significant difference (*p* < 0.05) using Tukey's multiple comparison test following one-way ANOVA.

To immortalize the gonadal somatic cells in medaka, the primary culture cells of a *mis (*−*3.1-kb)-GFP* transgenic testis (Figures 4A,B) were fused with an immortalized cell line, OLHE-131, using the PEG method. After being cultured for 1 week, 784 individual GFP-positive cells were picked and re-cultured in 96-well plates. After transferring five times, three immortalized hybridoma lines, FOT-01, FOT-02, and FOT-03 were established (Figure 4C). These hybridomas were morphologically similar to OLHE-131 and lost GFP fluorescence. Investigation of the proliferative potency of these lines revealed that although FOT-01 and FOT-02 had a higher proliferative potential than FOT-03, this potential was still significantly lower than that of OLHE-131 (Figure 4D). These results show that the method of cell fusion by PEG could be suitable for the establishment of immortalized hybridomas of medaka gonadal somatic cells.

### Analysis of Hybridoma Gene Expression

To investigate whether the immortalized hybridomas express the gonadal somatic cell markers, we examined germ cell and gonadal somatic cell markers using the hybridomas cultured for 3 weeks. qRT-PCR showed that the germ cell marker *dead end (dnd)* (32) was not expressed in any of the cell lines examined in this study (Figure 4E). Conversely, the Sertoli cell marker *mis* was expressed weakly in FOT-01 and FOT-02, and strongly in OLHE-131 (Figure 4F). Sertoli cell markers [*gonadal soma-derived growth factor (gsdf)* (38), *follicle-stimulating hormone receptor (fshr)* (25), *Mullerian inhibiting substance receptor type 2 (misr2)* (31)], Leydig cell markers [*3b-hydroxysteroid dehydrogenase (3b-hsd)* *(**39**), luteinizing hormone receptor (lhr)* (25)] and the nuclear receptors (*sf1, lrh1*) were expressed in FOT-02, but not in FOT-01 or FOT-03 (Figures 4G–M).

### PGC and Hybridoma Co-cultivation

**Figure 5:**
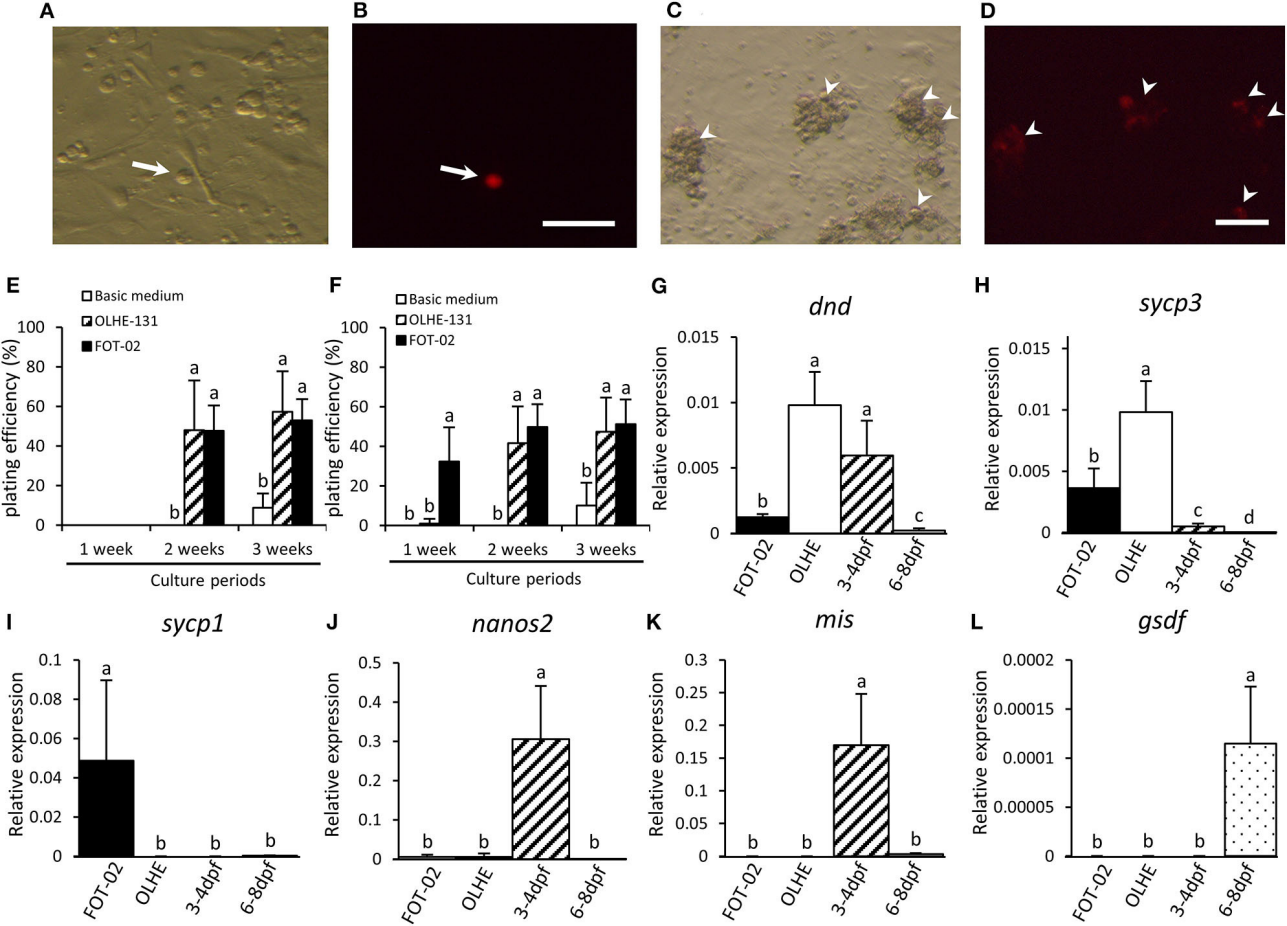
Characteristics of PGCs co-cultured with FOT-02 or OLHE-131 cells. DsRed fluorescence in a PGC dissociated from 6 to 8 dpf embryo in bright field **(A)** and in dark field **(B)**. Non-DsRed expressing cells are feeder cells or other somatic cells. DsRed fluorescence in colonizing PGCs co-cultured with FOT-02 for 1 week in bright field **(C)** and in dark field **(D)**. White arrow: DsRed-positive PGC; White arrowhead: DsRed-positive colonizing PGCs. Scale bar: 100 μm. PGC plating efficiency from 3 to 4 dpf **(E)** or 6–8 dpf embryos **(F)**. The assay conditions compared co-culture with FOT-02 or OLHE-131, and culture under basic medium. The PGCs cultured with FOT-02 had significantly colonized within 1 week. Confirmation of mRNA expression in colonizing cultured PGCs and gonadal regions from 3 to 4 dpf and 6–8 dpf embryos by qRT-PCR. *dnd* **(G)** and *sycp3* **(H)** were expressed in the PGCs co-cultured with FOT-02 and OLHE-131; *sycp1* **(I)** was confirmed only in PGCs co-cultured with FOT-02; *nanos2* **(J)** was not expressed in colonizing cultured PGCs; *mis* **(K)** and *gsdf* **(L)** were expressed in the gonadal region only. FOT-02: PGCs co-cultured with FOT-02; OLHE: PGCs co-cultured with OLHE-131; 3–4 dpf: gonadal regions from 3 to 4 dpf embryos; 6–8 dpf: gonadal regions from 6 to 8 dpf embryos. Vertical bar: mean ± standard error of triplicates or more; a, b, c, d, and ab: significant difference (*p* < 0.05) using Tukey's multiple comparison test following one-way ANOVA.

We confirmed that the established hybridoma FOT-02 can be used for *in vitro* PGC cultivation by co-culturing with *olvas-DsRed* transgenic medaka-derived PGCs (Figures 5A–D). PGCs were dissociated from 3 to 4 dpf embryos (germ cell migration stage), or 6–8 dpf embryos (germ cell proliferation stage) (Figures 5A,B) (40). The PGCs cultured in basic medium without the feeder cells formed a small number of colonies after 3 weeks of culture (Figures 5E,F). Conversely, the 3–4 dpf PGCs co-cultured with OLHE-131 or FOT-02 in basic medium proliferated and formed many colonies after 2 weeks of culture (Figure 5E). Moreover, the 6–8 dpf PGCs co-cultured with FOT-02—but not with OLHE-131—in basic medium, proliferated and formed significant colonies after 1 week of culture (Figures 5C–F).

### Analysis of Gene Expression in Cultured PGCs

To analyze the status of the cultured PGCs, we examined their expression of selected genes using the 6–8 dpf PGCs co-cultured with OLHE-131 or FOT-02 for 3 weeks. qRT-PCR showed that the germ cell marker *dnd*, and a meiotic marker *synaptonemal complex protein 3 (sycp3)* (41), were expressed in PGCs co-cultured with FOT-02 or OLHE-131 (Figures 5G,H). However, another meiotic marker, *synaptonemal complex protein 1 (sycp1)* (41), was confirmed only in PGCs co-cultured with FOT-02 (Figure 5I). A germline stem cell marker *nanos2* (42), and the gonadal somatic cell markers *mis* and *gsdf* , were not expressed in cultured germ cells (Figures 5J–L).

## Discussion

In this study, we have shown that medaka SF1 and LRH1 activate *mis* gene transcription, probably by binding to Ad4 sites on a 3.1-kb fragment of the *mis* promoter. Furthermore, GFP in *mis (*−*3.1-kb)-GFP* transgenic medaka, but not in *mis (*−*0.8-kb)-GFP* transgenic medaka, was strongly detected in gonadal somatic cells surrounding the germ cells in both sexes. Specifically, in adult gonads, GFP was localized in the Sertoli cells of the testis and in the granulosa cells of the ovary, which is similar to the expression pattern of endogenous medaka *mis* (31, 33). Thus, 3.1-kb of the *mis* promoter appears to be sufficient to drive the gonadal somatic cell-specific expression of *mis* in both sexes in medaka. In mice, it has been reported that 180 bp of the *MIS* promoter, which also includes an Ad4 site, is sufficient for expression specific to Sertoli cells in the embryonic testis and to granulosa cells of the postnatal ovary (19). Therefore, Ad4 sites may be important for gonadal somatic cell-specific expression of *mis* in both sexes across all vertebrates.

Next, to immortalize the gonadal somatic cells, we produced hybridoma cell lines by fusing Sertoli cells from a transgenic testis with a cell line derived from medaka hepatoma cancer. We used GFP fluorescence to screen and monoclone the products of cell fusions. In the middle of the cloning, we found that the fluorescence disappeared gradually in the cultured hybridomas, suggesting lose of *mis -GFP* vector from the genome in the cells during the culture. In contrast to previously reported medaka cell lines, which have not been clonal (12, 43, 44), we successfully established three monoclonal hybridomas. qRT-PCR showed that our established hybridomas, FOT-02 but not FOT-01 and FOT-03, expressed Sertoli cell markers as well as Leydig cell markers. Thus, this study reports the first establishment of a monoclonal hybridoma expressing both Sertoli and Leydig cell markers in medaka. Such characteristics are not found in the mouse Sertoli cell line TM4 or the mouse Leydig cell line TM3 (45). Previous study has demonstrated that the distinction between Sertoli cells and Leydig cells is regulated by Wilms' tumor gene 1 (*Wt1*), a zinc finger nuclear transcription factor, implying that these two cell types most likely originate from the same progenitor cells (46). Therefore, an established hybridoma, FOT-02 may be altered gene expression profiles by fusing normal Sertoli cells with the OLHE-131. Future study will need to investigate whether this hybridoma has characteristics of both Sertoli and Leydig cells.

Surprisingly, in the present study, we found that *mis* expression was significantly higher in OLHE-131 than the established hybridomas, nevertheless *lrh1* and *sf1* expression was not observed in the OLHE-131. Previously, it has been reported in human fibroblasts, hepatoma cells, and lymphoblasts that highly tissue-specific genes such as MIS, are expressed as spliced transcripts in non-specific tissues, suggesting that even in the absence of tissue-specific transcriptional factors, all promoters could be minimally active when ubiquitous transcriptional factors reach their cognate DNA elements (47). Therefore, *mis* expression may be induced by ubiquitous transcriptional factors in this OLHE-131 cells, although further investigation is needed.

To evaluate the functionality of this hybridoma, we co-cultured FOT-02 with PGCs. The PGCs from 6 to 8 dpf embryos significantly colonized in co-culture with FOT-02, after only 1 week of culture. Moreover, the PGCs from 3 to 4 dpf embryos colonized in co-culture with FOT-02 after 2 weeks. Conversely, when co-cultured with OLHE-131, the PGCs from either 3–4 or 6–8 dpf embryos only colonized after 2 weeks in co-culture. These results indicate that FOT-02 hybridoma cells, compared to OLHE-131 cells, can be useful feeder cells for inducing the early proliferation of PGCs in 6–8 dpf embryos. We also examined gene expression patterns in the cultured PGCs. qRT-PCR showed that the germ cell marker *dnd*, and a meiotic marker *sycp3*, were expressed in the PGCs co-cultured with OLHE-131 higher than in those with FOT-02; however, another meiotic marker, *sycp1* was confirmed only in PGCs co-cultured with FOT-02. Previous study shows that *sycp3* is expressed earlier than *sycp1* during medaka meiosis (41). Therefore, this suggests that the PGCs co-cultured with FOT-02 cells initiate meiosis earlier than those cultured with OLHE-131 cells.

In mice PGCs co-cultured with feeder cells, the presence of stem cell factor (SCF), leukemia inhibitory factor (LIF), and basic fibroblast growth factor (bFGF) is essential for long-term maintenance and proliferation *in vitro* (48). It has also been reported that the presence of glial cell-derived neurotrophic factor (GDNF) is essential for the maintenance of spermatogonia (4, 49). Conversely, zebrafish spermatogonial stem cells (SSC) colonized in co-culture with a Sertoli cell line ZtA6-12, express Sycp3 and differentiate into functional sperm (8, 50). Moreover, zebrafish SSC have been successfully maintained over long periods in the presence of GDNF, bFGF, insulin-like growth factor 1 (IGF-1), heparin, 2-mercaptoethanol, and dorsomorphin hydrochloride (9, 50). Judging from these results, the long-term maintenance of medaka PGCs, and their differentiation into functional sperm, may be feasible via co-culture with FOT-02 cells, and treatment with certain growth factors.

In summary, we have demonstrated that medaka SF1 and LRH1 activate *mis* transcription and that *mis (*−*3.1-kb)-GFP* transgenic medaka emit GFP fluorescence specifically in gonadal somatic cells in the gonads. Moreover, we successfully established a hybridoma, FOT-02, with gonadal somatic cell-like gene expression, by fusing GFP-expressing cells from *mis (*−*3.1-kb)-GFP* medaka with OLHE-131 cells. The PGCs picked from *olvas-DsRed* transgenic embryos at 6–8 dpf proliferated as early as 1 week after co-culturing with FOT-02, and these proliferated germ cells expressed the meiotic marker genes *sycp1* and *sycp3* by 3 weeks. These results provide the first evidence in teleosts that a successfully established gonadal somatic cell-derived hybridoma can induce both the proliferation and meiosis of germ cells.

## Data Availability Statement

The datasets presented in this study can be found in online repositories. The names of the repository/repositories and accession number(s) can be found in the article/Supplementary Material.

## Ethics Statement

The animal study was reviewed and approved by Animal Care and Use Committee of 3 Kumamoto University (Approval number: 30-022). Written informed consent was obtained from the owners for the participation of their animals in this study.

## Author Contributions

TKa and TKi obtained funding and designed the study and wrote the manuscript. TKa, HK, SH, TS, ES, KM, TY, SI, and TKi performed the experiments and collected the data. All authors contributed to the article and approved the submitted version.

## Conflict of Interest

TKa, HK, and SI were employed by the company ARK Resource Co., Ltd. The remaining authors declare that the research was conducted in the absence of any commercial or financial relationships that could be construed as a potential conflict of interest.
