# Supplementary material for: Transcriptional Regulation of Müllerian Inhibiting Substance (MIS) and Establishment of a Gonadal Somatic Cell Line Using mis-GFP Transgenic Medaka (Oryzias latipes)
Source: Front Endocrinol (Lausanne). 2020 Sep 29;11:578885. doi: 10.3389/fendo.2020.578885 (PMC7550650; doi:10.3389/fendo.2020.578885)
Supplement: Supplementary file 1 [file Data_Sheet_1.PDF]

Supplementary Table 1. List of primers used in this study.

Gene	Accession No.	Primer sequences
GFP	FJ172221.1	For, 5'- AACGGCCACAAGTTCAGCGT
		Rev, 5'- TCCTCCTTGAAGTCGATGC
sf1	AB016834	For, 5'- GTTGGGAGACAAAGCTCACG
		Rev, 5'- TTCTCAGCGCACGTGTACCT
lrh1	DQ132803	For, 5'- ATGCTGCCCAAAGTCGAGAC
		Rev, 5'- CGTAGTGATACCCTGAAACC
dmy	AB071534	For, 5'- GTCAAGCAGAGTTTGAGAG
		Rev, 5'- CATTTGTGACCAACATCTTCTG
mis	AB214971	For, 5'- AAGCAGCGGAGAGTCACTTC
		Rev, 5'- TTGATGTTGGCGCTCTGCGG
dnd	GQ184560	For, 5'- AGGTGGTGAACTTGGAGCGG
		Rev, 5'- CTGCAGCAGCTCCTCCTGC
gsdf	AB525390	For, 5'- ATGTCTTTGGCACTCATTGTC
		Rev, 5'- ACTGTCGCATGACACAGAGG
misr2	DQ499644	For, 5'- GCAGGTTGTGGGACAAGGAC
		Rev, 5'- GAGCAATCCCAGCATGCCTC
3b-hsd	AB426890	For, 5'- ATGTCTCTGAGAGGTGATGTG
		Rev, 5'- AAGTCGGAGTCYCTGATGTC
fshr	AB526237	For, 5'- ATCTACTTGACCTACCGCAAGC
		Rev, 5'- ACTGAGAGTATTGGCCAAGGAG
lhr	AB526238	For, 5'- ACCGCATCACGGCCATACAG
		Rev, 5'- GGTCAAGGAGGAGACGGATG
nanos2	AB437936	For, 5'- GGTGCAAACAACTGTGGATG
		Rev, 5'- CTTGCAGAAGCGGCAGTAAT
sycp1	AB207974	For, 5'- CCAGTACGACCGGATGCTTG
		Rev, 5'- CAGTCAGTTCCTTCTGCACG
sycp3	AB162905	For, 5'- GTCAAGCAAAAGCACCTGG
		Rev, 5'- GTAGTTTCTTCTGCAGCGTGG
ef1α	AB013606	For, 5'- TGAGATGGGCAAGGGCTCCT
		Rev, 5'- GCTGGGTTGTAGCCGATCTT
mis-Kpn(3000)	AB214971	For, 5'- TGGGTACCGCTGCACGCGCTCCTGCTGG
mis-Kpn(800)		For, 5'- ACCGGTACCAAAGCAGGACTGTAACTGTGA
mis-Kpn(200)		For, 5'- TGCGGTACCTCCTCTGTTATGTGGACGTTG
mis-Xho		Rev, 5'- TGCTCGAGTGCTGTCTGAGCGGCTCTCTGG
mis-Bam		Rev, 5'- ATGGATCCGTGCTGTCTGAGCGGCTCTCTG
sf1-Eco	AB016834	For, 5'- GCTGAATTCATGTTGGGAGACAAAGC
sf1-Xho		Rev, 5'- GTGCTCGAGTCACACGCACGCTCTC
lrh1-Eco	DQ132803	For, 5'- GGGAATTCATGCTGCCCAAAGTCGAG
lrh1-Xho		Rev, 5'- TTCTCGAGTTTAAGCTCTTTTAGCGTGC
